# Peculiarities of the Presentation of the Encephalitogenic MBP Peptide by HLA-DR Complexes Providing Protection and Predisposition to Multiple Sclerosis

**DOI:** 10.32607/actanaturae.11008

**Published:** 2021

**Authors:** A. E. Mamedov, I. N. Filimonova, I. V. Smirnov, A. A. Belogurov

**Affiliations:** Shemyakin-Ovchinnikov Institute of Bioorganic Chemistry Russian Academy of Sciences, Moscow, 117997 Russia; Institute of Fundamental Medicine and Biology, Kazan (Volga) Federal University, Kazan, 420008 Russia; Lomonosov Moscow State University, Moscow, 119991 Russia

**Keywords:** multiple sclerosis, HLA-DR, protective allele, risk allele, MBP peptide, viral peptide

## Abstract

Predisposition to multiple sclerosis (MS), a chronic autoimmune disease of the central nervous system, is due to various factors. The genetic component is considered one of the most important factors. HLA class II genes contribute the most to the development of MS. The HLA-DRB1*15 allele group is considered one of the main genetic risk factors predisposing to MS. The group of HLA-DRB1*01 alleles was shown to have a protective effect against this disease in the Russian population. In this work, we compared the binding of the encephalitogenic fragment of the myelin basic protein (MBP) to two HLA-DR complexes that provide protection against and predisposition to MS: HLA-DR1 (HLA-DRB1*0101) and HLA-DR15 (HLA-DRB1*1501), respectively. We found that the myelin peptide MBP_88-100_ binds to HLA-DR1 at a rate almost an order of magnitude lower than the viral peptide of hemagglutinin (HA). The same was true for the binding of MBP_85-97_ to HLA-DR15 in comparison with viral pp65. The structure of the C-terminal part of the peptide plays a key role in the binding to HLA-DR1 for equally high-affinity N-terminal regions of the peptides. The IC_50_ of the myelin peptide MBP_88-100_ competing with viral HA for binding to HLA-DR1 is almost an order of magnitude higher than that of HA. As for HA, the same was also true for the binding of MBP85-97 to HLA-DR15 in comparison with viral pp65. Thus, autoantigenic MBP cannot compete with the viral peptide for binding to protective HLA-DR1. However, it is more competitive than viral peptide for HLA-DR15.

## INTRODUCTION

The human leukocyte antigen (HLA) genes encode proteins that can bind to and present antigenic peptides. Therefore, they play a critical role in the immune response to pathogens and autoimmunity [1]. Binding of antigenic peptides to HLA class II molecules leads to the formation of binary peptide–HLA complexes. These complexes are presented on the surface of antigen- presenting cells (APCs) and recognized by CD4 T cell receptors [2]. Newly synthesized HLA proteins are protected against aggregation by the invariant chain [3]. In the endosomal compartment, the invariant chain is partially degraded, thus leaving the CLIP peptide in the binding groove [4, 5]. CLIP can be further exchanged for antigenic peptides, which form as a result of antigen processing in endosomes. The exchange process is catalyzed by the HLA-DM protein [6]. The peptide–HLA complex is transported next to the APC surface for recognition by CD4 T cells. The mechanisms of peptide presentation by HLA class II molecules are well known [7]. However, it remains unclear how the formation and presentation of autoantigen–HLA complexes lead to autoimmune reactions, and there is substantial interest in the topic. Thus, the identification of the autopeptide– HLA complexes associated with autoimmune responses may provide a clue to our understanding of the pathogenesis of autoimmune diseases [8, 9, 10].

Multiple sclerosis (MS) is a chronic autoimmune disease of the central nervous system which is characterized by inflammation, demyelination, and neurodegeneration [11]. The nature of the genetic predisposition to MS is complex and depends on a combination of multiple genetic and epigenetic factors, not to mention environmental factors [12]. The genes in the HLA region are considered to contribute substantially to the risk of MS [13]. Certain alleles of the highly polymorphic HLA class II gene *DRB1 *appear to be a significant genetic determinant in the pathology of MS and can affect both predisposition and resistance to the disease [14]. The HLA-DRB1*1501 allele and the haplotype associated with it (DQA1*0102, DQB1*0602, DRB1*1501, and DRB5*0101) have been known as universal risk factors for MS since the 1970s. An analysis of the association of HLA with MS in Northern European populations revealed the groups of HLA-*DRB1 *alleles (DRB1*03, *01, *10, *11, *14, *08) in positive or negative correlation with the risk of the disease [15]. Furthermore, the autoantigenic peptides presented by the risk alleles have been identified. HLA-DRB1*1501 binds a fragment of myelin basic protein (MBP), the encephalitogenic peptide MBP_85-99_ [8], while HLA-DRB5*0101 presents the MBP_86-105_ peptide [10]. The CD4 T cell clones that recognize these peptide–HLA complexes associated with the disease have been identified as well [16 , 17, 18].

It was previously shown in a representative cohort of ethnic Russian patients with MS and conditionally healthy individuals that the group of HLA-DRB1*01 alleles is associated with MS resistance, while HLA-DRB1*15 alleles are positively associated with the disease. An analysis of the interaction of proteins encoded by the HLA-DRB1*1501 risk allele and the protective allele HLA-DRB1*0101 with the MBP library demonstrated that both proteins can bind myelin peptide MBP_81-104_ with similar affinity [19]. However, it is unclear how binding of the same myelin fragment provides protection in the case of one allele and predisposition to the disease in the case of the other allele. Therefore, the aim of this study was to analyze the kinetic characteristics of the interaction of peptide MBP_81-104_ with the protective HLA-DR1 and predisposing HLA-DR15 in patients with MS, as well as to compare them to their viral antigenic determinants.

## EXPERIMENTAL

**Expression and purification of proteins**

Recombinant proteins HLA-DR1 (the product of the HLA-DRA1*0101 and HLA-DRB1*0101 genes), HLA-DR15 (the product of the HLA-DRA1*0101 and HLA-DRB1*1501 genes), and HLA-DM were obtained using the method described previously [20]. The CLIP peptide (PVSKMRMATPLLMQA) was covalently bound to the N-terminus of the β-chains of HLA-DR1 and HLA-DR15 via a linker with a thrombin cleavage site, at which the peptide was cleaved for further experiments (1 h, 20 U/mg, 25°C). Proteins were concentrated in PBS and stored at 4°C.

Peptides fused to thioredoxin were designed and obtained using a previously constructed MBP epitope library [21]. Genetic constructs coding for HA, pp65, myelin peptides (MBP_88-100_ and MBP_85-97_), MBP with point mutations (V86A, V87A, F89A, and F90A), and chimeric peptides (HA-MBP, MBP-HA, and pp65-MBP) were obtained by PCR using the MBP epitope library as a template. The protein constructs were presented by peptides fused to the C-terminus of bacterial thioredoxin through a flexible linker (SGGGG)3S carrying His-tags for purification. The construct carrying only thioredoxin with a linker (TRX) was used as a negative control. All thioredoxin-fused peptides were obtained using the method described previously [21]. The peptides were chemically biotinylated with EZ-Link Sulfo-NHS-LC-biotin (Thermo Fisher Scientific) at a molar ratio of 1 : 20 for 30 min at 25°C. Proteins were concentrated in PBS and stored at –20°C.

**ELISA for analyzing HLA-DR peptide binding**

**Fig. 1:**
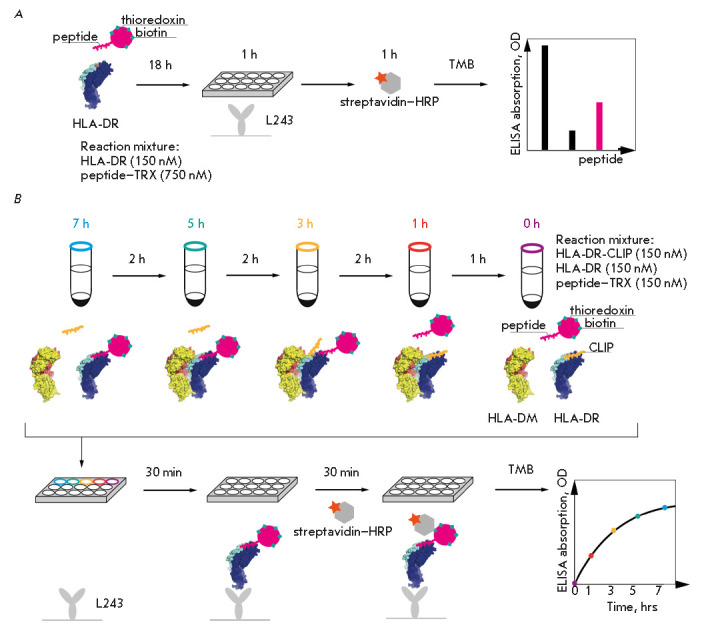
Schematic representation of ELISA for the binding of HLA-DR peptides (*A*) and kinetics of peptide loading onto HLA-DR (*B*). Each time point is marked with color. L243 – immobilized monoclonal antibodies to HLA-DR

Biotinylated peptide MBP_81-104_ and its variants with point mutations (V86A, V87A, F89A, and F90A) (750 nM) were incubated in 50 μL of PBS with CLIPbound HLA-DR (HLA-DR1 or HLA-DR15) (150 nM) at 37°C for 18 h (*Fig. 1A*). Thioredoxin with a linker (TRX) was used as a negative control. DR–eptide complexes were then added to anti-HLA-DR antibodies (L243) immobilized on the plate and blocked by PBS containing a 2% skim milk powder. The biotinylated peptide bound to HLA-DR was quantified using horseradish peroxidase-conjugated streptavidin.

In a competitive assay, biotinylated HA and pp65 peptides (150 nM) were incubated with the corresponding HLA-DR (HLA-DR1 or HLA-DR15) (150 nM) in the presence of either non-biotinylated HA, pp65, myelin (MBP_88-100_ and MBP_85-97_) or chimeric (HA-MBP, MBP-HA, and pp65-MBP) peptides at concentrations of 1,000; 500; 250; 125; 62.5; 31.2; 15.6; and 7.8 nM in 50 μL of PBS at 37°C for 18 h. Experiments were carried out in triplicate.

**ELISA for analyzing the kinetics of peptide loading onto HLA-DR**

The corresponding HLA-DR bound to CLIP (HLA-DR1 or HLA-DR15) (150 nM) was incubated in the presence of HLA-DM (150 nM) in 50 μL of citrate buffer (50 mM sodium citrate, 150 mM NaCl, pH 5.3) with either biotinylated HA, pp65, myelin (MBP_88-100_ and MBP_85-97_) or chimeric (HA-MBP, MBP-HA, and pp65-MBP) peptides (150 nM) at 37°C for 7, 5, 3, 1, and 0 h (*Fig. 1B*). For each time point, the experimental system was mixed separately every 2 h starting from the longest incubation time (7 or 5 h), after which all time points were simultaneously added to the plate. ELISA was performed as described above, with the only difference being that the time of incubation of the reaction mixtures in the plate with streptavidin was reduced to 30 min. Experiments were carried out in triplicate. The kinetic curves were analyzed using the Enzyme Kinetics module of the SigmaPlot software (Sigma-Aldrich). The binding curves were fitted using a nonlinear leastsquares fit to the Langmuir binding model describing a 1 : 1 binding stoichiometry.

## RESULTS AND DISCUSSION

**Determining the MBP_81-104_ epitopes recognized by HLA-DR1 and HLA-DR15**

**Fig. 2:**
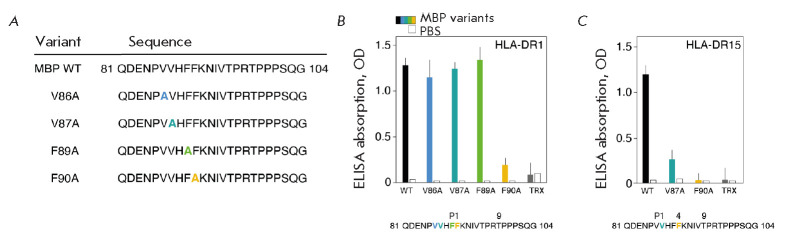
(*A*) Sequences of peptide MBP81-104 and its variants with point amino acid substitutions to alanine. Point substitutions are indicated with different colors. (*B, C*) Binding of peptide MBP81-104 and its variants (750 nM) carrying point amino acid substitutions to alanine with CLIP-bound HLA-DR1 (*B*) and HLA-DR15 (*C*) (150 nM). Column colors correspond to the colors of point substitutions. White bars represent the background signal (PBS). Thioredoxin (TRX) with a linker was used as a negative control. Standard deviation is presented

We have compared the kinetic characteristics of the interaction between the encephalitogenic fragment of the myelin basic protein MBP_81-104_ and human major histocompatibility complex class II proteins, namely MS-protective HLA-DR1 and MS-predisposing HLA-DR15 [19], as well as their antigenic determinants of viral origin. In order to conduct our analysis, it was first necessary to determine the binding epitope within MBP_81-104_ recognized by HLA-DR1. Alanine scanning (substitution of hydrophobic and aromatic residues with alanine starting from the N-terminus of the peptide (*Fig. 2A*)) of MBP_81-104_ revealed a Phe90 residue acting as a hydrophobic anchor at P1 (*Fig. 2B*). This led us to suggest that the pockets P6/P7 and P9 in HLA-DR1 that are bound to MBP_81-104_ are occupied by Thr95, Pro96, and Thr98 residues. Identification of the MBP_81-104_ epitope responsible for binding to HLA-DR15, in which Val87 and Phe90 are located at positions P1 and P4, respectively [8], was confirmed using the corresponding mutant forms of MBP_81-104_ (*Fig. 2C*).

**Comparison of the kinetics of MBP peptide loading onto HLA-DR1 and HLA-DR15**

**Fig. 3:**
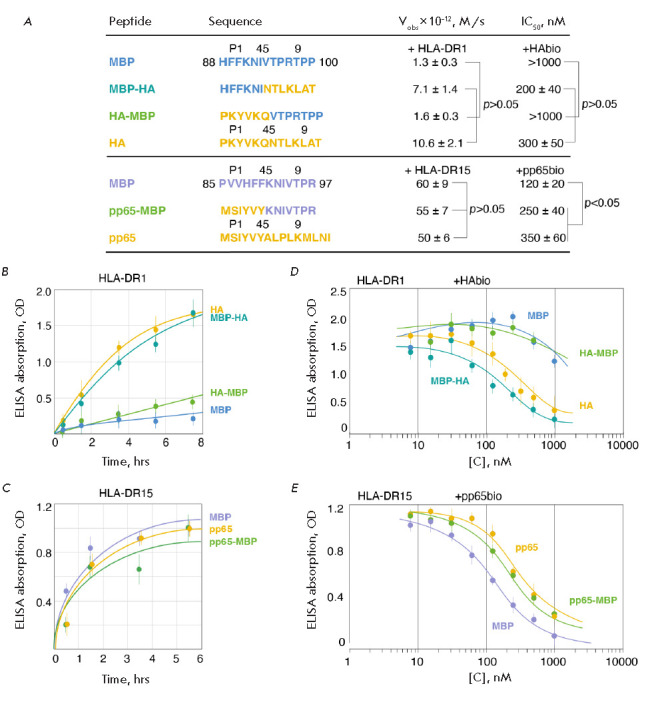
(*A*) Sequences of the peptides MBP_88-100_, MBP-HA, HA-MBP, HA, MBP_85-97_, pp65-MBP, and pp65. Various parts of the chimeric peptides, as well as the positions of the amino acid residues P1/4/5/9, are indicated with different colors. For each of the peptides, the initial rates of interaction with the corresponding CLIP-bound HLA-DR1 or HLA-DR15, as well as the IC_50_ values in competition with the HAbio and pp65bio peptides, are shown. (*B, C*) Kinetics of binding of the biotinylated peptides MBP_88-100_, MBP-HA, HA-MBP, and HA (150 nM) to CLIP-bound HLA-DR1 (150 nM) (*B*), as well as of the biotinylated peptides MBP_85-97_, pp65-MBP, and pp65 (150 nM) to CLIP-bound HLA-DR15 (150 nM) (*C*) in the presence of HLA-DM (150 nM). (*D, E*) Competitive interaction of HLA-DR1 (150 nM) and HLA-DR15 (150 nM) with the biotinylated peptides HAbio (150 nM) (*D*) and pp65bio (150 nM) (*E*), respectively, in the presence of increasing concentrations (7.8 nM – 1 μM) of non-biotinylated peptides MBP_88-100_, MBP-HA, HA-MBP, and HA (*D*), as well as MBP_85-97_, pp65-MBP, and pp65 (*E*) in the presence of HLA-DM (150 nM). Standard deviation and p-values are presented

At the next stage, we studied the kinetics of the binding of HLA-DR1 to the peptides HA_306-318_, MBP_88-100_, and their chimeric constructs MBP-HA and HA-MBP in the presence of HLA-DM, which accelerates the rate of CLIP exchange for the peptide under study (*Fig. 3B*). HA is a fragment of the influenza virus hemagglutinin, a classic viral antigenic determinant for HLA-DR1 [22]. For comparison with the HLA-DRB1*1501 risk allele, binding curves for the interaction of HLA-DR15 with peptide pp65_109-123_ (which is a fragment of a cytomegalovirus protein), a HLA-DR15 viral determinant [23], myelin peptide MBP_85-97_, and chimeric construct pp65-MBP were also obtained (*Fig. 3C*). It is important to note that, in chimeric peptides, the boundary between the N- and C-terminal regions of the constituent peptides lay between the amino acid residues at positions P4 and P5 (*Fig. 3A*).

Viral peptide HA is known to possess a high affinity to the peptide-binding groove of HLA-DR1 [24]. Therefore, the kinetic curve for the interaction between peptide HA and HLA-DR1 reaches a plateau after 8 h (*Fig. 3B*). At the same time, the myelin peptide MBP_88-100_ binds to HLA-DR1 at a rate almost an order of magnitude lower than that of the viral peptide. Thus, we can assume that protective HLA-DR1 kinetically distinguishes between the exogenous viral and endogenous myelin antigens. The chimeric peptide HA-MBP, which contains the N-terminal region of HA (306–311) and the C-terminal part of MBP (94–100), binds to HLA-DR1 at a low rate. This rate is similar to the kinetics of interaction with MBP_88-100_. However, in the case of the chimeric MBP-HA peptide composed of the N-terminal region of MBP (88–93) and the C-terminal region of HA (312–318), the binding rate is very high. The same is true in the case of binding of natural viral HA. Based on these findings, we can conclude that the kinetic parameters of binding of chimeric peptides to HLA-DR1 indicate the importance of the C-terminal region for an efficient interaction with HLA-DR1 with equally high-affinity N-terminal peptide regions. The N-terminal parts of the fragments under study contain the main anchor in the P1 binding pocket: the aromatic residues Tyr308 and Phe90 in the case of HA and MBP, respectively. One can assume that it is the presence of Pro96 in the C-terminal region of MBP_88-100_ and chimeric HA-MBP peptides that changes the peptide position in the binding groove. This happens due to the inherent conformational rigidity of proline and impairs any interaction of the peptide with the P7 binding pocket. In the HA and MBP-HA peptides, the P7 pocket contains a hydrophobic Leu314 within the C-terminal region, which favors binding.

An analysis of the allele responsible for the risk of MS demonstrated that viral, myelin, and chimeric peptides bind to HLA-DR15 at similar rates (*Fig. 3C*). In contrast to HLA-DR1, the key element for peptide binding in HLA-DR15 is the P4 pocket, where aromatic amino acid residues fit ideally. The hydrophobic P1 pocket is second in significance. Therefore, the efficiency of binding of the viral, myelin, and chimeric peptides can be due to the amino acid residues favoring an interaction of peptides with the peptide-binding groove in HLA-DR15. These amino acids are located in the pockets P1 and P4, which are important for peptide loading: Ile111 and Tyr114 in the case of pp65, as well as Val87 and Phe90 in the case of MBP. Despite the fact that the viral peptide pp65 also contains proline in the P7 pocket at the C-terminal region, it does not decrease the efficiency of peptide interaction with the peptide-binding groove. This happens because the pockets P6/P7/P9 play a lesser role than P4 in the case of HLA-DR15. A discrepancy in the rate of interaction of HA with HLA-DR1 and pp65 with HLA-DR15 (about fivefold) can be attributed to differences in the structure of the pockets of these HLA-DR complexes and the presence of anchor residues in the corresponding peptides (*Fig. 3A*).

Differing rates of loading of various peptides of exogenous and endogenous nature onto MS-protective HLA-DR1 and MS-predisposing HLA-DR15 may be an indication that the kinetic component (rather than the thermodynamic one) plays a greater role in the interaction between the MHC II complex and the antigens.

**Comparison of the competitiveness of MBP for binding to HLA-DR1 and HLA-DR15**

Taking into account the fact that myelin peptide binds to both HLA-DR1 and HLA-DR15, albeit at different rates, the question of whether it can compete for binding with high-affinity viral antigens remained open. To clarify this issue, we conducted some experiments to study the competitive ability of HA, myelin peptide MBP_88-100_, as well as the chimeric peptides HA-MBP and MBP-HA to bind HLA-DR1 in the presence of viral HAbio (*Fig. 3D*). An analysis was also performed for the interaction of pp65, myelin peptide MBP_88-100_, and chimeric peptide pp65-MBP with HLA-DR15 in the presence of viral pp65bio (*Fig. 3E*). The kinetic data indicate that HA and chimeric peptide MBP-HA can effectively compete with viral HAbio for HLA-DR1. Moreover, addition of these peptides significantly decreases the ELISA signal starting from a concentration of 30 nM. On the contrary, addition of myelin peptide MBP88-100 and chimeric peptide HA-MBP insignificantly reduces the ELISA signal, which is observed only at high concentrations (starting from 300 nM) (*Fig. 3D*). The IC_50_ values of these peptide pairs differ by almost an order of magnitude, which indicates that myelin peptide MBP88-100 cannot effectively compete with viral HA for binding to HLA-DR1 (*Fig. 3A*). In the case of HLA-DR15, the decline in the ELISA signal in the competitive reactions with MBP_85-97_ and pp65 starts at a pp65bio concentration of 30 nM (*Fig. 3E*). This is similar to the interaction between the HA peptide and HLA-DR1. At the same time, the IC_50_ of peptide MBP_85-97_ is approximately threefold lower than that of pp65. Thus, unlike MBP_88-100_, MBP_85-97_ is even more competitive than viral peptide (*Fig. 3A*).

## CONCLUSIONS

According to our findings, it is fair to assume that, in contrast to HLA-DR15, it is unlikely that a fragment of the myelin basic protein is presented as its complex with HLA-DR1 on the surface of antigen-presenting cells at the density required for the activation of the T cell response. Apparently, the protective properties of the HLA-DRB1*0101 allele are associated with the ability of its protein product HLA-DR1 to distinguish kinetically between myelin and exogenous peptides. Meanwhile, HLA-DR15, which is associated with the risk of MS, can efficiently present the MBP fragment even when competing with exogenous peptides such as viral pp65. Our data suggest that the same encephalitogenic myelin fragment can be presented at a completely different rate depending on the HLA-DR allele. In other words, the immunogenicity of myelin components in patients with MS may be largely determined by genetic predisposition due to carriage of a specific HLA-DR allele rather than by their accessibility to immune cells.

## Abbreviations

APC: antigen-presenting cell
ELISA: enzyme-linked immunosorbent assay
HLA: human leukocyte antigen
MBP: basic myelin protein
MS: multiple sclerosis
PBS: phosphate-buffered saline
TRX: thioredoxin.
